# Homozygous *LMNA* p.R582H pathogenic variant reveals increasing effect on the severity of fat loss in lipodystrophy

**DOI:** 10.1186/s40842-020-00100-9

**Published:** 2020-07-14

**Authors:** Utku Erdem Soyaltin, Ilgin Yildirim Simsir, Baris Akinci, Canan Altay, Suleyman Cem Adiyaman, Kristen Lee, Huseyin Onay, Elif Arioglu Oral

**Affiliations:** 1 Division of Endocrinology and Metabolism, Department of Internal Medicine, Ege University, Izmir, Turkey; 2 Division of Endocrinology and Metabolism, Department of Internal Medicine, Dokuz Eylul University, Izmir, Turkey; 3 Division of Metabolism, Endocrinology and Diabetes (MEND), Department of Internal Medicine, University of Michigan, Ann Arbor, MI USA; 4 Department of Radiology, Dokuz Eylul University, Izmir, Turkey; 5 Division of Genetics, Metabolism & Genomic Medicine, Department of Pediatrics, Ann Arbor, MI USA; 6 Department of Medical Genetics, Ege University, Izmir, Turkey

**Keywords:** *LMNA*, Lamin A, Generalized lipodystrophy, Homozygous, Partial lipodystrophy

## Abstract

**Background:**

Classical heterozygous pathogenic variants of the lamin A/C (*LMNA*) gene cause autosomal dominant familial partial lipodystrophy type 2 (FPLD2). However, recent reports indicate phenotypic heterogeneity among carriers of *LMNA* pathogenic variants, and a few patients have been associated with generalized fat loss.

**Case presentation:**

Here, we report a patient with a lamin A specific pathogenic variant in exon 11, denoted *LMNA* (c.1745G > A; p.R582H), present in the homozygous state. Fat distribution was compared radiographically to an unrelated heterozygote *LMNA* p.R582H patient from another pedigree, a healthy female control, a series of adult female subjects with congenital generalized lipodystrophy type 1 (CGL1, *n* = 9), and typical FPLD2 (*n* = 8). The whole-body MRI of the index case confirmed near-total loss of subcutaneous adipose tissue with well-preserved fat in the retroorbital area, palms and soles, mons pubis, and external genital region. This pattern resembled the fat loss pattern observed in CGL1 with only one difference: strikingly more fat was observed around mons pubis and the genital region. Also, the p.R582H *LMNA* variant in homozygous fashion was associated with lower leptin level and earlier onset of metabolic abnormalities compared to heterozygous p.R582H variant and typical FPLD2 cases. On the other hand, the heterozygous *LMNA* p.R582H variant was associated with partial fat loss which was similar to typical FPLD2 but less severe than the patients with the hot-spot variants at position 482.

**Conclusions:**

Our observations and radiological comparisons demonstrate an additive effect of *LMNA* pathogenic variants on the severity of fat loss and add to the body of evidence that there may be complex genotype-phenotype relationships in this interesting disease known as FPLD2. Although the pathological basis for fat loss is not well understood in patients harboring pathogenic variants in the *LMNA* gene, our observation suggests that genetic factors modulate the extent of fat loss in *LMNA* associated lipodystrophy.

## Background

Lipodystrophy is a heterogeneous group of diseases characterized by near-total or partial lack of subcutaneous adipose tissue. The common clinical presentation of congenital generalized lipodystrophy (CGL) is the near-complete absence of adipose tissue and generalized muscular appearance which can be recognized at birth. Four major subgroups of CGL have been described, and are inherited in an autosomal recessive manner. They are CGL1 (biallelic acylglycerol-3-phosphate O-acyltransferase 2 [*AGPAT2*] pathogenic variants), CGL2 (biallelic Berardinelli-Seip congenital lipodystrophy type 2 [*BSCL2*] pathogenic variants), CGL3 (biallelic caveolin 1 [*CAV1*] pathogenic variants), and CGL4 (biallelic polymerase 1 and transcript release factor [*PTRF*] pathogenic variants) [1].

Monoallelic pathogenic variants of the *LMNA* gene, encoding lamin A/C nuclear-envelope proteins, cause familial partial lipodystrophy type 2 (FPLD2), an autosomal dominant disorder with distinct phenotypic heterogeneity [2]. Typical *LMNA* pathogenic variants associated with FPLD2 are located in exon 8 at the amino acid position 482. Patients with FPLD appear normal at birth but lose subcutaneous fat predominantly from extremities after puberty, resulting in prominent, well-defined musculature in these areas [3].

Here, we report a patient with lamin A specific homozygous *LMNA* p.R582H pathogenic variant in exon 11. Fat distribution was compared to an unrelated heterozygous *LMNA* p.R582H carrier from another pedigree, and also a series of subjects with CGL1 and typical FPLD2. Although homozygosity for the *LMNA* p.R582H variant was associated with generalized fat loss pattern, heterozygosity for the p.R582H variant revealed partial fat loss which was similar to typical FPLD2. Comparison of patients with biallelic and monoallelic *LMNA* p.R582H variants revealed a more severe phenotype with more pronounced fat loss in the patient with biallelic pathogenic variant.

## Case presentation

A 29-year-old Turkish woman was referred from the in vitro fertilization clinic for the management of her diabetes and hypertriglyceridemia. Her menstrual periods had been irregular since menarche at age 12. She had four pregnancies, three of them resulted in second-trimester fetal loss and one in ectopic pregnancy.

She had first noted well-defined muscles in her arms and legs around puberty. Subcutaneous fat progressively had disappeared from her limbs, gluteal region, trunk and abdomen in a generalized pattern. Although the patient was unable to define a clear period of time when subcutaneous fat disappeared, she reported fat loss since childhood. At the age of 17, she was diagnosed with diabetes and metformin (2 g per day) monotherapy was initiated. Three years later, her triglyceride level was found to be 1900 mg/dL, and fenofibrate was started. Although metformin helped her keep glucose levels in the target range for a while, her HbA1c gradually increased above 7%. Her triglyceride levels, on the other hand, remained significantly elevated despite treatment with fenofibrate.

**Fig. 1:**
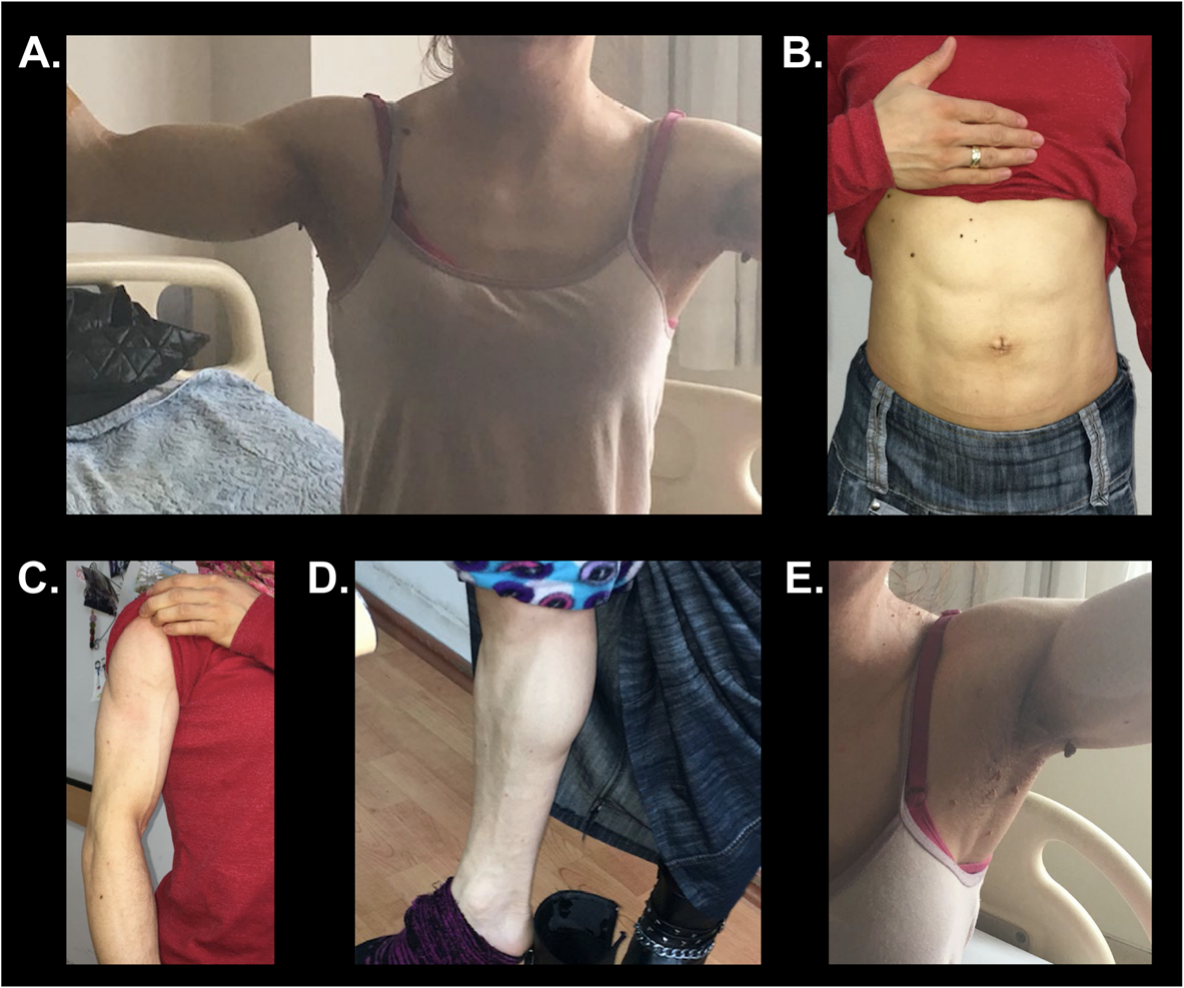
Patient pictures showing generalized fat loss. **a**: Near-total lack of adipose tissue and muscular appearance in the trunk. Arms are very muscular. Note that there is no fat accumulation in the neck. In contrast, subcutaneous fat is lost over shoulders and in the upper trunk. **b**: Subcutaneous fat is lost in the abdomen. Muscular appearance is remarkable. **c**: Arms are muscular with visible vessels and no subcutaneous fat. **d**: Subcutaneous fat is lost in the distal legs. Legs are muscular with prominent veins. **e**: Acanthosis nigricans and skin tags in the armpits associated with severe insulin resistance

Physical examination revealed generalized fat loss (Fig. 1a and b) and prominent muscles in her upper (Fig. 1c) and lower extremities (Fig. 1d). Facial fat was decreased. She had well-preserved subcutaneous fat in the mons pubis area and around her external genital region. Her body mass index (BMI) was 19.2 kg/m^2^. She had acanthosis nigricans in the axillary regions (Fig. 1e), and the liver was palpable 2 cm below the costal margin.

Her leptin level was 0.4 ng/mL. Lab tests are presented in Table 1. Her genetic testing was negative for pathogenic *AGPAT2* and *BSCL2* variants but revealed a homozygous p.R582H (c.1745G > A) pathogenic variant of the *LMNA* gene. She has a brother, and her parents are alive; however, family screening could not be performed because of lack of consent.

**Table 1:** Comparison of clinical characteristics and laboratory results of patients

	Homozygous *LMNA* p.R582H (*n* = 1)	Heterozygous *LMNA* p.R582H (*n* = 1)	CGL1 (*n* = 9)	Typical FPLD2 (*n* = 8)
Age (years)	29	48	25 (18–34)	49 (32–62)
BMI (kg/m^2^)	19.20	27.28	19.53 (16.61–22.20)	23.22 (19.53–26.20)
The age when lipodystrophy was diagnosed (years)	29	33	16 (1–31)	49 (12–60)
The age when diabetes developed (years)	17	32	14 (6–25)	33 (21–51)
Oral antidiabetic use (Yes/No)	Yes	Yes	8/1	9/9
Insulin (Yes/No)	No	No	9/0	5/3
Complications of diabetes (Yes/No)	No	No	8/1	7/1
The age when hypertriglyceridemia was first detected (years)	20	33	16 (6–26)	37 (20–51)
Lipid medication (Yes/No)	Yes	Yes	9/9	6/2
History of pancreatitis (Yes/No)	No	Yes	3/6	1/7
The age when hepatic steatosis was first detected (years)	29	33	17 (6–29)	35 (20–51)
HbA1c (%)	7.9	6.2	10.4 (7.6–11.7)	8.5 (6.3–11)
Leptin (ng/mL)	0.4	9.81	0.38 (< 0.1–0.85)	1.53 (0.94–7.42)
HOMA-IR^†^	14.1	7.66	9.06 (3.98–78.57)	8.12 (5.63–14.43)
AST (IU/L)	27	23	23 (11–145)	17 (11–32)
ALT (IU/L)	30	32	24 (15–114)	19 (9–43)
HDL Cholesterol (mg/dL)	22	38	28 (15–36)	33 (21–46)
Triglyceride (mg/dL)	1600	169	597 (72–2083)	443 (196–1358)
Creatinine (mg/dL)	0.69	0.71	1.21 (0.37–2.29)	0.75 (0.50–1.50)
Urinary protein excretion (mg/day)	41.8	11	245 (< 5–15,200)	173 (< 5–3210)

^†^HOMA-IR was calculated as fasting insulin (microU/L) x fasting glucose (nmol/L)/22.5. Leptin normal range for adult females (BMI: 22): 3.3–18.3 ng/mL. ALT: alanine aminotransferase AST: aspartate aminotransferase. *AGPAT2* pathogenic variants in CGL1 patients are IVS5–2 A > C (c.662-2A > C), *n* = 1; p.C48X (c.144C > A), *n* = 3; p.E229X (c.685G > T), *n* = 2; p.E172K (c.514G > A), *n* = 2; p.R68X (c.202C > T), *n* = 1; and p.D180PfsX5 (c.538_539delGA), *n* = 1. *LMNA* pathogenic variants in typical FPLD2 patients are p.R482Q (c.1445G > A), *n* = 4; and p.R482W (c.1444C > T), *n* = 4. Lipid medications include fenofibrate 250–267 mg/day. Oral antidiabetic use includes metformin (1–2 g/day), DDP4 inhibitors (sitagliptin 100 mg/day, vildagliptin 100 mg/day), and pioglitazone (15–30 mg/day). Values were reported as median (range) in the CGL1 and FLPD2 groups

Her fat distribution was assessed by the whole-body MRI which was acquired by using a 1.5-T MRI system with a 6 multichannel body coil (Gyroscan Intera, release 8.1; Philips Medical Systems, Best, the Netherlands), and compared to an unrelated 48-year-old female with a monoallelic *LMNA* p.R582H pathogenic variant. Also, whole-body MR images were compared to the images available from a 28-year-old healthy woman and a series of adult female patients with CGL1 (*n* = 9) and typical FPLD2 (*n* = 8).

**Fig. 2:**
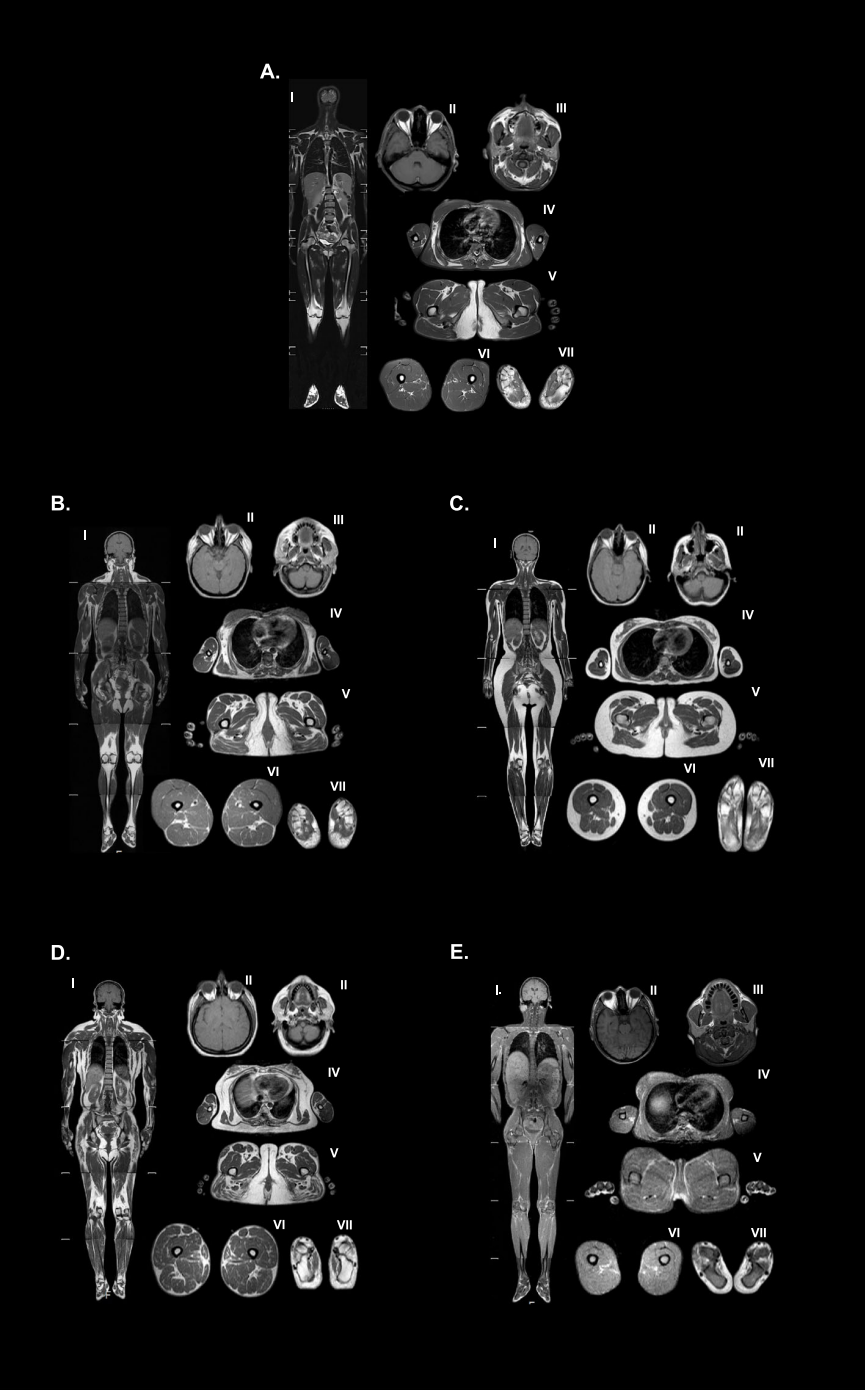
Comparison of fat distribution. The whole-body MRI confirms generalized fat loss in the patient who had a homozygous pathogenic variant in the *LMNA* gene. Adipose tissue is well preserved around mons pubis and external genital region similar to heterozygous *LMNA* R582H patient and typical FPLD2 patients while fat tissue loss is noted in a generalized pattern in the scalp, mammary gland, abdomen visceral/subcutaneous, and extremities. Fluid like signal is detected in the bone marrow. Supraclavicular subcutaneous fat was preserved, but the amount of fat was significantly decreased in contrast to heterozygous *LMNA* R582H and typical FLPD2. The liver was steatotic and diffusely enlarged. Fat loss is partial in heterozygous *LMNA* p.R582H carrier (Fig. 2b) affecting the limbs, abdomen, breasts and the lower part of the body which is similar to typical FPLD2, although more subcutaneous fat was observed in the upper part of the trunk, over the shoulders, and head and neck (Fig. 2b and d). Retroorbital fat is preserved in all patients. **a**: Fat distribution in the patient with homozygous *LMNA* pathogenic variant, R582H; **b**: Fat distribution in the patient with heterozygous *LMNA* pathogenic variant, p.R582H; **c**: Fat distribution in a healthy control (28 years old, female); **d**: Fat distribution in a 30-year-old female with typical FPLD2 caused by heterozygous *LMNA* pathogenic variant p.R482W (c.1444C > T); **e**: Fat distribution in a 30-year-old female with the classical CGL1 phenotype caused by homozygous *AGPAT2* pathogenic variant p.C48X (c.144C > A). In each panel I. Whole-body T1-weighted imaging; II. Retroorbital, axial T1 weighted- imaging; III. Head and neck, axial T1 weighted-imaging; IV. Trunk, axial T1 weighted-imaging; V. Pelvic region, axial T1 weighted-imaging, VI. Upper leg, axial T1 weighted imaging; VII. Sole, axial T1-weighted imaging

The study revealed near-total loss of subcutaneous adipose tissue with preserved fat in the retroorbital area and palms and soles in our patient, resembling fat loss pattern observed in CGL1 (Fig. 2a and e). However, adipose tissue was very well preserved around mons pubis and external genital region (Fig. 2a), which is an unlikely finding in CGL1. Also, supraclavicular subcutaneous fat was preserved, but the amount of fat was decreased in contrast to typical FLPD2. The liver was diffusely enlarged, and hepatic steatosis was noted. On the other hand, fat loss was partial in the monoallelic *LMNA* p.R582H carrier (Fig. 2b) which was similar to typical FPLD2, although more subcutaneous fat was observed in the upper part of the body (Fig. 2b and d).

## Discussion and conclusions

Lipodystrophy phenotypes associated with *LMNA* typically segregate as monoallelic pathogenic alleles in families, leading to an autosomal dominant pattern of FPLD [3]. Recent reports indicate a distinct phenotypic heterogeneity among carriers of *LMNA* pathogenic variants. Also, several groups reported generalized fat loss in carriers of *LMNA* pathogenic variants. A series of patients with heterozygous *LMNA* p.T10I pathogenic variant has been linked to GL-associated progeroid syndrome [4, 5]. Additionally, a recent report described juvenile-onset CGL caused by a heterozygous missense *LMNA* pathogenic variant p.R571S affecting lamin C [6].

Our patient had near-total fat loss caused by homozygosity of the p.R582H *LMNA* pathogenic variant. Heterozygosity for the same *LMNA* pathogenic variant was associated with partial fat loss, suggesting a cumulative effect with more severe fat loss in the homozygous subject. Also, homozygosity of the p.R582H *LMNA* variant was associated with lower leptin level and earlier onset of metabolic abnormalities compared to heterozygous p.R582H and typical FPLD2. On the other hand, metabolic abnormalities seem to be less severe with homozygosity of p.R582H *LMNA* compared to CGL1 presumably due to later onset of near-total fat loss. Biallelic pathogenic variants in *LMNA* associated lipodystrophy are very rare. Biallelic pathogenic variants of *LMNA* have been previously linked to autosomal recessive axonal neuropathy (Charcot-Marie-Tooth disorder type 2), mandibuloacral dysplasia type A, and autosomal recessive Hutchinson-Gilford progeria syndrome [7]. Wiltshire et al. [8] previously reported that homozygosity for *LMNA* R482Q resulted in a combination of generalized lipodystrophy and Emery Dreifuss muscular dystrophy. Andre et al. [9] reported genotype-phenotype correlations in a large pedigree from Reunion Island in which there were patients with both homozygous and heterozygous *LMNA* T655fsX49 pathogenic variant. Homozygous patients had an earlier onset of disease, more severe subcutaneous fat loss, and lower leptin levels. Also, they presented with more overlapping syndromes with severe cardiac phenotypes such as lower left ventricular ejection fraction, coronary heart disease and high-degree conduction disorder.

The *LMNA* gene encodes lamins A and C by alternative splicing within exon 10. Lamin A and C form polymers at the nuclear lamina which maintains the structural meshwork of the nuclear envelope, therefore, regulating the stability of the nucleus [7]. Although classical exon 8 defects in FPLD2 affect both lamins A and C, pathogenic variants within the exon 11 affect lamin A only. We and others previously reported several pedigrees with heterozygous missense *LMNA* p.R582H pathogenic variant in exon 11 [2, 3]. Very recently, Montenegro et al. [10] reported clinical and metabolic features of four patients from the same family with *LMNA* p.R582C pathogenic variant, three homozygous and one in the heterozygous state that present with three distinct lipodystrophic phenotypes. Similar to our patient, one of these family members with homozygous *LMNA* p.R582C pathogenic variant had generalized lipodystrophic features; however, no imaging could be performed as the patient passed away. Interestingly, shortly after submitting our paper, we came across another case report of a homozygous *LMNA* variant associated with generalized lipodystrophy phenotype which was released online very recently. In this new independent report, Patni et al. [11] reported two sisters with near-generalized loss of subcutaneous fat caused by homozygous *LMNA* p.R545H (c.1634G > A) pathogenic variant. These patients had metabolic abnormalities associated with insulin resistance such as diabetes mellitus, extreme hypertriglyceridemia, and hepatic steatosis, and also early onset intellectual disability, short stature, clinodactyly, joint contractures, leiomyoma of the uterus, and cataracts in childhood.

In conclusion, lipodystrophy is a very heterogeneous disease with a current classification based on clinical and morphometric features. Our data together with emergent data from other groups suggest that biallelic pathogenic variants of *LMNA* cause a more severe lipodystrophy phenotype. Therefore, we recommend screening for the *LMNA* gene in patients who may present with even generalized lipodystrophy phenotype. Although the pathological basis for an additive genetic variation on the severity of fat loss in lipodystrophy is not known, our observation suggests that genetic factors modulate the extent of fat loss in *LMNA* associated lipodystrophy.

## Data Availability

The datasets analyzed during this study are included in this published article.

## Abbreviations

*AGPAT2*: Acylglycerol-3-phosphate O-acyltransferase 2
*BSCL2*: Berardinelli-Seip congenital lipodystrophy type 2
BMI: Body mass index
*CAV1*: Caveolin 1
CGL: Congenital generalized lipodystrophy
*LMNA*: Lamin A/C
MRI: Magnetic resonance imaging
FPLD2: Partial lipodystrophy type 2
*PTRF*: Polymerase 1 and transcript release factor

## References

[1] Akinci B, Meral R, Oral EA (2018). Phenotypic and genetic characteristics of Lipodystrophy: pathophysiology, metabolic abnormalities, and comorbidities. Curr Diab Rep.

[2] Garg A, Vinaitheerthan M, Weatherall PT, Bowcock AM (2001). Phenotypic heterogeneity in patients with familial partial lipodystrophy (dunnigan variety) related to the site of missense mutations in Lamin a/c gene. J Clin Endocrinol Metab.

[3] Akinci B, Onay H, Demir T, Savas-Erdeve S, Gen R, Simsir IY, Keskin FE, Erturk MS, Uzum AK, Yaylali GF (2017). Clinical presentations, metabolic abnormalities and end-organ complications in patients with familial partial lipodystrophy. Metabolism.

[4] Mory PB, Crispim F, Kasamatsu T, Gabbay MA, Dib SA, Moises RS (2008). Atypical generalized lipoatrophy and severe insulin resistance due to a heterozygous LMNA p.T10I mutation. Arq Bras Endocrinol Metabol.

[5] Hussain I, Patni N, Ueda M, Sorkina E, Valerio CM, Cochran E, Brown RJ, Peeden J, Tikhonovich Y, Tiulpakov A (2018). A novel generalized Lipodystrophy-associated Progeroid syndrome due to recurrent heterozygous LMNA p.T10I mutation. J Clin Endocrinol Metab.

[6] Patni N, Xing C, Agarwal AK, Garg A (2017). Juvenile-onset generalized lipodystrophy due to a novel heterozygous missense LMNA mutation affecting Lamin C. Am J Med Genet A.

[7] Ho R, Hegele RA (2019). Complex effects of laminopathy mutations on nuclear structure and function. Clin Genet.

[8] Wiltshire KM, Hegele RA, Innes AM, Brownell AK (2013). Homozygous Lamin a/C familial lipodystrophy R482Q mutation in autosomal recessive Emery Dreifuss muscular dystrophy. Neuromuscul Disord.

[9] Andre P, Schneebeli S, Vigouroux C, Lascols O, Schaaf M, Chevalier P (2015). Metabolic and cardiac phenotype characterization in 37 atypical Dunnigan patients with nonfarnesylated mutated prelamin a. Am Heart J.

[10] Montenegro RM, Costa-Riquetto AD, Fernandes VO, Montenegro A, de Santana LS, Jorge AAL, Karbage L, Aguiar LB, Carvalho FHC, Teles MG (2018). Homozygous and Heterozygous Nuclear Lamin A p.R582C Mutation: Different Lipodystrophic Phenotypes in the Same Kindred. Front Endocrinol (Lausanne).

[11] Patni N, Hatab S, Xing C, Zhou Z, Quittner C, Garg A. A novel autosomal recessive lipodystrophy syndrome due to homozygous LMNA variant. J Med Genet. 2019. 10.1136/jmedgenet-2019-106395 PMC8376205 31857427

